# Some Eye-Openers

**Published:** 1894-10

**Authors:** 


### SOME EYE-OPENERS.

Eyes half open showing whites. Agar.
— — closed. Op.
— — — balls turned upward. Op.
— — — during sleep. Op., Pod., Stram., Sulph., Ipec., Ant-tart., Arsen.
— — — Epilepsy. Op.
— — — opened with difficulty. Lyc.
— — — lightly closed. Lauroc.
— — — during sleep. Pod.
— — — — labor. Lyc.
— — — especially children. Pod.
— — — during sleep. Stram.
— — —. Amyg.
— — — hydrocephalus. Apis.
— — — —. Art-vulg.

Eyes half closed, scarlatina. Carbol-ac.
— — — pneumonia. Ant-t.
— — — during sleep. Sulph.
— — — — —. Ipec.
— -- — brain troubles. Hellebor.
— — — or wide open. Hydroceph., Hellebor.
— — — Terebinth., Lach.

